# Characterization of Gold Nanorods Conjugated with Synthetic Glycopolymers Using an Analytical Approach Based on spICP-SFMS and EAF4-MALS

**DOI:** 10.3390/nano11102720

**Published:** 2021-10-15

**Authors:** Milica Velimirovic, Alessia Pancaro, Robert Mildner, Panagiotis G. Georgiou, Kristof Tirez, Inge Nelissen, Christoph Johann, Matthew I. Gibson, Frank Vanhaecke

**Affiliations:** 1 Department of Chemistry, Atomic & Mass Spectrometry–A&MS Research Group, Campus Sterre, Ghent University, Krijgslaan 281-S12, 9000 Ghent, Belgium; frank.vanhaecke@ugent.be; 2 Flemish Institute for Technological Research (VITO), Boeretang 200, 2400 Mol, Belgium; alessia.pancaro@vito.be (A.P.); kristof.tirez@vito.be (K.T.); inge.nelissen@vito.be (I.N.); 3 Advanced Optical Microscopy Centre and Biomedical Research Institute, Hasselt University, 3590 Diepenbeek, Belgium; 4 Wyatt Technology Europe GmbH, Hochstrasse 12a, 56307 Dernbach, Germany; Robert.Mildner@wyatt.eu (R.M.); cjohann@wyatt.eu (C.J.); 5 Department of Chemistry, University of Warwick, Gibbet Hill Road, Coventry CV4 7AL, UK; P.Georgiou@warwick.ac.uk (P.G.G.); M.I.Gibson@warwick.ac.uk (M.I.G.); 6 Warwick Medical School, University of Warwick, Gibbet Hill Road, Coventry CV4 7AL, UK

**Keywords:** gold nanorods, gold nanorods conjugated with synthetic glycopolymers, high-resolution single-particle inductively coupled plasma-mass spectrometry, electrical asymmetric-flow field-flow-fractionation combined with multi-angle light scattering

## Abstract

A new comprehensive analytical approach based on single-particle inductively coupled plasma-sector field mass spectrometry (spICP-SFMS) and electrical asymmetric-flow field-flow-fractionation combined with multi-angle light scattering detection (EAF4-MALS) has been examined for the characterization of galactosamine-terminated poly(N-hydroxyethyl acrylamide)-coated gold nanorods (GNRs) in two different degrees of polymerization (DP) by tuning the feed ratio (short: DP 35; long: DP 60). spICP-SFMS provided information on the particle number concentration, size and size distribution of the GNRs, and was found to be useful as an orthogonal method for fast characterization of GNRs. Glycoconjugated GNRs were separated and characterized via EAF4-MALS in terms of their size and charge and compared to the bare GNRs. In contrast to spICP-SFMS, EAF4-MALS was also able of providing an estimate of the thickness of the glycopolymer coating on the GNRs surface.

## 1. Introduction

Gold nanorods (GNRs) have promising biomedical applications, mainly because of their unique optical properties dominated by the localized surface plasmon resonance (LSPR) phenomenon: their anisotropic shape causes a splitting of their optical absorption bands into two peaks, corresponding to the transverse and longitudinal plasmon resonances. The longitudinal resonance peak position is highly shape and size dependent and is highly sensitive to refractive index changes in the local environment, such as those caused by binding of biomolecules to the rod surface [1]. Moreover, it is shifted from the visible to the near-infrared (NIR) region with increasing aspect ratio (length/width) [2,3] where biological tissues have the highest optical transparency. This makes the GNRs appropriate for in vivo and in vitro applications [4]. They have shown promising results in cancer diagnostics (using GNRs for enhancing two-photon excited luminescence) [5] and treatment (using Plasmonic photothermal therapy and Photodynamic therapy) [6,7,8,9,10,11]. Furthermore, they have been investigated for gene therapy and drug delivery applications [12,13]. GNRs-based LSPR sensors have been applied in the context of in vitro diagnostics involving a wide variety of disease-specific biomarker targets [14,15,16].

The utilization of GNRs for biomedical applications requires an appropriate functionalization to provide chemical stability and biocompatibility, and to recognize target molecules in a biological environment. It has been demonstrated that the chemical nature, the linker length, as well as the grafting density of the polymer coating used, has a dramatic impact on the outcomes of glyco-nanoparticle biosensing performance, enabling aggregative versus non-aggregative outputs and providing a dose-dependent optical response even in complex biological environments [16]. However, the synthesis and characterization of GNRs with well-defined sizes, shapes, and bioconjugated surfaces remains an important challenge. Techniques employed by different research groups for the characterization of gold nanoparticles, as well as GNRs and bioconjugated GNRs are (high-resolution) transmission electron microscopy (HRTEM), Scanning transmission electron microscopy/Energy-dispersive X-ray spectroscopy/High-angle annular dark-field imaging (STEM/EDXS/HAADF), Fourier transform infrared spectroscopy (FTIR), UV-Visible spectroscopy (UV-Vis), dynamic light scattering (DLS) and ζ-potential measurements [17,18,19]. Nanoparticle tracking analysis (NTA) and differential centrifugal sedimentation (DCS) have also been used for GNRs characterization [20].

**Figure 1:**
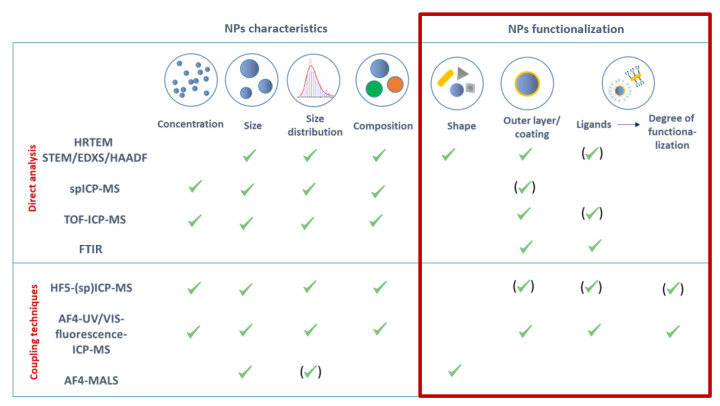
Overview of analytical techniques potentially applicable for the characterization of gold nanorods conjugated with synthetic glycopolymers.

As these techniques have their own limitations, complementary analytical techniques, such as single-particle inductively coupled plasma-mass spectrometry (spICP-MS), single particle time-of-flight ICP-MS (spTOF-ICP-MS), hollow-fiber flow field-flow fractionation and asymmetric-flow field-flow-fractionation combined with multi-angle light scattering detection (HF5-MALS and AF4-MALS, respectively) may present interesting alternatives (Figure 1). 

In recent years, spICP-MS has emerged as a reliable tool allowing one to distinguish between ionic and particulate signals, and providing information on particle number concentration, particle size and size distribution [21]. However, its use for the complex nanoparticle (NP) samples with different functionalization is limited. The major advantages of spICP-MS over other techniques for NP characterization and quantification are the minimal sample preparation, the superior sensitivity and the element specificity [22]. However, the technique also exhibits drawbacks, such as the limited multi-element capabilities or the complete absence thereof when using quadrupole-based ICP-MS systems, which are the most common type of mass analyzers in ICP-MS instrumentation. However, the new generation of high-resolution spICP-MS (spICP-SFMS) instrumentation provides much faster detection capabilities in comparison to other types [23]. Despite its excellent sensitivity, detection power in terms of minimum NP size is still lacking and highly material-dependent (for most nanoparticles in the range of 10–20 nm or even higher). Although considerable progress has been made, spICP-MS still needs further development with numerous opportunities for optimization, e.g., in the context of GNRs [24]. 

Finally, field-flow-fractionation separation (e.g., asymmetric flow field flow fractionation, hollow-fiber flow-field-flow fractionation-HF5) represents a powerful analytical tool providing high-resolution separation of particles in the size range of 1 nm to several micrometers. When combined with an adequate detection approach, it provides information on particle size, size distribution, shape, and chemical composition (stoichiometry) of the particles studied [25]. In addition, electrical asymmetric-flow field-flow-fractionation hyphenated to a multi-angle light scattering detector (EAF4-MALS) combines high-resolution separation with surface charge (Zeta potential) measurement [26,27]. As such, it presents a promising tool for characterization of GNRs conjugated with synthetic glycopolymers. 

The main aim of this work is to propose a new set of analytical tools (methods) for physicochemical characterization of GNRs conjugated with short and long synthetic glycopolymers for biosensing of lectins in terms of particle size, coating thickness and/or surface charge in comparison with the bare GNRs based on the use of spICP-SFMS and EAF4-MALS. 

## 2. Materials and Methods

### 2.1. Chemicals and Materials

All chemicals used in this study were of analytical purity. For spICP-SFMS, ultra-pure water (18.2 MΩ cm) was obtained from a Milli-Q system (Millipore, Burlington, MA, USA). High-purity (optima grade) 14 M HNO_3_ and 12 M HCl were obtained from Fisher Chemical (Loughborough, UK). Appropriate dilutions of 1000 mg L^−1^ Au Certipur^®^ (Merck, Darmstadt, Germany) in 2 M HNO_3_ traceable to SRM from NIST H(AuCl₄) were used for method development and spICP-SFMS calibration purposes. Suspensions of spherical gold nanoparticles (GNPs) with a diameter of 27.6 (NIST SRM 8012) and 56.0 nm (NIST SRM 8013) (National Institute of Standards and Technology NIST, Gaithersburg, MD, USA) [28,29] were used to determine the transport efficiency (TE) based on the particle size method [30].

Citrate-stabilized GNRs (further referred as GNRs) of 10 nm width and 38 nm length were purchased from Nanopartz (Loveland, USA/Canada). Monomer N-hydroxyethyl acrylamide (97%, HEA), 2-(dodecylthiocarbonothioylthio)-2-methylpropionic acid pentafluorophenyl ester (98%, PFP-DMP) and D-(+)-galactosamine were all purchased from Sigma-Aldrich (Steinheim am Albuch, Germany). Poly(N-hydroxyethyl acrylamide) (PHEA) was synthetized by photo-initiated reversible addition-fragmentation chain transfer (RAFT) polymerization in two lengths, corresponding to a different degree of polymerization of 35 and 60; then they were functionalized with D-(+)-galactosamine achieving the glycopolymers Gal-PHEA35 and Gal-PHEA60, as previously described [20].

Pure water (15 MΩ cm) for EAF4-MALS carrier solution preparation was obtained from an Elix 3 Advantage system (Merck, Darmstadt, Germany). Sodium nitrate, ≥99.5% purity was purchased from Merck, Darmstadt, Germany. Precut 5 kDa cutoff polyether sulfone (PES) membranes were obtained from Wyatt Technology Europe, Dernbach, Germany [31]. For effective channel height calibration, 20 nm gold nanoparticles (BAM-N004, Bundesanstalt für Materialforschung und-prüfung, Berlin, Germany) were used.

### 2.2. Preparation of Glycoconjugated GNRs

Preparation of glycoconjugated GNRs (GNR-Gal-PHEA35 and GNR-Gal-PHEA60) has been previously described by Pancaro et al. [20]. Briefly, poly(N-hydroxyethyl acrylamide) (PHEA) was synthesized by photo-initiated RAFT polymerization and modified with galactosamine (Gal) [16]. RAFT installs sulfur-containing end-groups which have high affinity for gold surfaces [32,33,34] and enables installation of a glycan conjugation unit at the opposing end-group [35,36]. The glycopolymers used in this study have two different degrees of polymerization (DP = 35, 60) determined by proton nuclear magnetic resonance analysis in methanol-d4. Moreover, narrow monomodal molecular weight distributions determined by size exclusion chromatography were observed with low dispersity values (Đ_M_ ≤ 1.3) indicating a controlled photo-polymerization (Synthetic Method S1, ESI).

**Figure 2:**
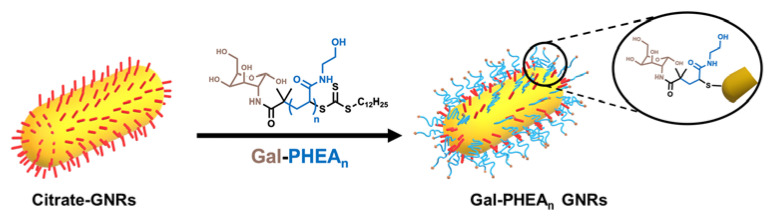
Functionalization of citrate-stabilized gold nanorods using Gal-PHEA telechelic homopolymers of different chain lengths (n). Note, RAFT agent cleavage can occur during functionalization depending on the excess used, but does not affect GNR immobilization.

Citrate-GNRs were functionalized with 4 mg of each glycopolymer (Figure 2) dissolved in 200 µL of water and mixed by pipetting with 800 µL of GNRs suspension at 10 OD. After 1 h of incubation at room temperature in the dark, the particles were sonicated for 1 min using an ultrasonic bath at 40 kHz (Branson 1800 series CPX1800H), centrifuged at 12,000 RCF and 20 °C for 15 min using a Sigma 3-30KS centrifuge. Subsequently, the supernatant was removed. This was followed by three cycles of resuspension in 1 mL of water, centrifugation and decanting. The particles were finally resuspended in 1 mL of water and stored in polypropylene graduated tubes at 4 °C until use. The samples were characterized using UV-Vis, ζ-potential, DLS, DCS and NTA confirming the successful attachment of the glycopolymers to the particle surface (Table S1, ESI). Retention of the pentafluoro phenyl end-group during polymerization and its displacement at the α-terminus [37] after galactosamine installation was confirmed via fluorine-NMR and FTIR measurements [16].

### 2.3. Instrumental Analysis

#### 2.3.1. UV-Visible Spectroscopy

UV-Vis absorption spectra were acquired at room temperature (25 °C) using a CLARIOstar Plus spectrophotometer (BMG LABTECH, Cary, NC, USA). The absorbance spectra were recorded in a wavelength range of λ = 400–1000 nm with 1 nm resolution and 30 s of plate shaking at 100 RPM applied before measurement. Results were smoothed using a Savitzky-Golay filter (order 4, window width 31). Peak maxima were determined from the zero crossings of the derivative of the smoothed data. All measurements were performed at least in triplicate (n ≥ 3).

#### 2.3.2. Nanoparticle Tracking Analysis (NTA)

A NanoSight NS500 instrument (Malvern Panalytical, Worcestershire, UK) in scatter mode with a laser output of 75 mW at 532 nm (green) and sCMOS camera (camera level set at 15) was used. The samples were analyzed in duplicate at 25 °C and three videos of 60 s were recorded (1499 frames with 25 frames per second) for each sample. The number of particles/frame ranged from 30 to 90 for the GNR samples, and none were detected in the buffer control. The samples were diluted to 10^8^–10^9^ particles per mL in Milli-Q water. For calibration, 100 nm polystyrene (PS) microspheres were used. The mode was derived from a particle number concentration-based size distribution using the NTA software version 3.0.

#### 2.3.3. Dynamic Light Scattering (DLS)

Dynamic light scattering (DLS) was measured on a Zetasizer ZS (Malvern Panalytical, Worcestershire, UK). Measurements were carried out using a 4 mW He-Ne 633 nm laser module operating at 25 °C at an angle of 173° (back scattering), and results were analyzed using Malvern DTS 7.03 software. All determinations were repeated in triplicate with at least three measurements recorded for each run.

#### 2.3.4. Differential Centrifugal Sedimentation (DCS)

Differential centrifugal sedimentation (DCS) was performed to assess the binding of the glycopolymers on the GNR surface by measuring the peak size distribution of the particles. For this purpose, a CPS DC24000 disc centrifuge was used with an 8–24% (*w*/*w*) sucrose gradient and a rotation speed of 24,000 RPM. Before each run, well-defined, polyvinyl chloride latex beads (239 nm) were used as calibration standard to ensure accuracy of the measurements. All the measurements were performed at least in duplicate (n ≥ 2). The settling of particles is shape-dependent; for the GNRs, application of a ‘non-sphericity factor’ of 2.85 in the CPS software provided a light scattering function close to the correct scattering function for the particles.

A model to analyze data for protein shell-coated particles was developed by Monopoli et al. [38]; it enables the biocorona thickness to be estimated from DCS data. Briefly, the particles are treated as a high-density metallic core with a lower-density shell of biomolecules. A core-shell mathematical model can be used to calculate the shell thickness from the shift in particle sedimentation time before and after functionalization, knowing the size and density of the core nanoparticle. Moreover, it is important to point out that the binding of biomolecules onto the GNRs’ surface increases the particles’ size, but lowers their overall density. The DCS analysis assumes a constant particle density, so over-estimating the particle density brings about an under-estimation of the particle size [39]. For this reason, the binding of polymers or biomolecules to the GNRs results in an apparent decrease in the particle size reported by CPS.

Characteristics of the bare GNRs, GNR-Gal-PHEA35 and GNR-Gal-PHEA60 based on DCS (Figure S1) are given in Table 1.

#### 2.3.5. spICP-SFMS

All samples were analyzed with a Nu Attom ICP-MS (Nu Instruments Ltd., Wrexham, UK). This instrument is equipped with a double-focusing sector field mass spectrometer, with forward (Nier-Johnson) geometry. In single-particle mode, a single *m/z* value is monitored (i.e., the magnetic field and the acceleration voltage are fixed). For the measurement of GNRs, the instrument was operated at low resolution (R∼300). Samples were introduced using a conventional sample introduction system, consisting of a glass concentric nebulizer with a nominal uptake rate of 300 μL min^−1^ (self-aspirating) mounted onto a quartz cyclonic spray chamber. The actual uptake rate was determined by weighing the mass of water before and after transfer of sample into the system by the peristaltic pump for 10 min. spICP-SFMS was used in the time-resolved analysis (TRA) mode, with a dwell time of 40 µs and an acquisition time of 60 s (Table 2).

#### 2.3.6. EAF4-MALS

Particle fractionation and particle sizing was carried out using an Eclipse EAF4 Separation System (Wyatt Technology, Santa Barbara, CA, USA) and an Agilent 1260 high-performance liquid chromatograph (HPLC) unit equipped with a quaternary pump with integrated degasser and vialsampler (Agilent Technologies, Santa Clara, CA, USA). A DAWN 18-angle MALS detector operating with a 660 nm laser (Wyatt Technology, Santa Barbara, CA, USA) was coupled to the fractionation system and the signal was monitored under a 90° angle. A precut 5 kDa cutoff polyether sulfone (PES) membrane and a 350 μm height spacer were introduced inside the Mobility Channel. The carrier solution consisted of a 0.5 mM sodium nitrate aqueous solution (pH = 8.42). For separation, the cross-flow was maintained at 0.5 mL min^−1^ for 30 min. The detailed separation settings for the EAF4 experiments are summarized in Table 3.

The duration of a representative EAF4 run was 45 min with the data acquisition interval set to 0.5 s. For mobility measurements, an amperage of −0.1 mA (bottom electrode negatively charged) was applied during separation. Data were collected and analyzed in VISION^®^ software (Wyatt Technology, Santa Barbara, CA, USA). The dilution factor with pure water was chosen for each sample individually, aiming to have a final Au concentration of approximately 2–3 mg L^−1^. Typically, 300 µL of bare GNRs suspension or 30 µL of glycoconjugated GNRs suspension were injected per run to obtain similar signal intensities for all samples. Sample recovery was calculated based on the 90° light scattering (LS) signal peak area compared to flow-through injections of the samples without focus step and without cross-flow or electrical field applied during elution.

## 3. Results

### 3.1. Characterization of GNRs and Glycoconjugated GNRs by spICP-SFMS

**Figure 3:**
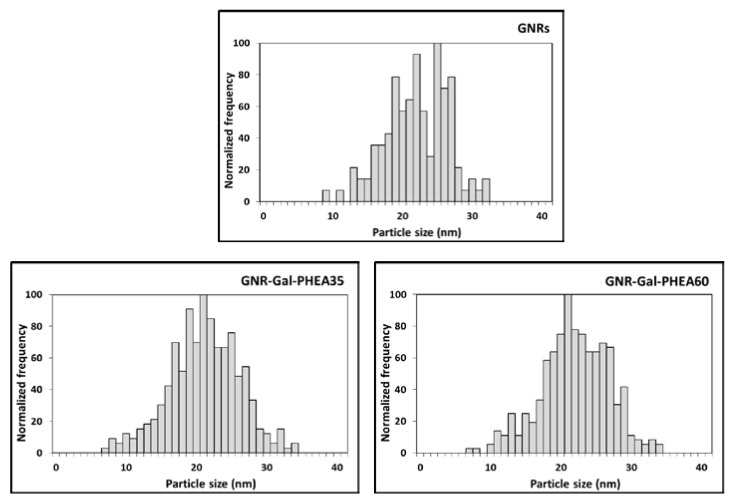
Particle size distributions of GNRs, GNR-Gal-PHEA35 and GNR-Gal-PHEA60 as obtained using spICP-SFMS.

The spherical equivalent diameter and particle size distribution data of GNRs, GNR-Gal-PHEA35 and GNR-Gal-PHEA60 (Figure 3, Table 4) were determined in order to exa-mine the possibility of using spICP-SFMS to derive the thickness of the glycopolymers layer bound onto the GNRs surface.

For the primary particles, spICP-SFMS measurements provided a similar size as did the TEM analysis reported by Pancaro et al. [20] shown in Figure S2. In addition, spICP-SFMS provided the particle number concentration (Table 4). We observed that the particle size result for the bare GNRs (21.0 ± 0.5 nm) was similar to that for the GNRs bond with Gal-PHEA35 on the surface (21.0 ± 0.4 nm). In case of binding with long glycopolymers (GNR-Gal-PHEA60), the spherical equivalent diameter increased to 22.0 ± 0.0 nm. As a result, it is clear that these data do not provide a reliable assessment of the thickness of the glycopolymer layer conjugated to the GNRs surface. Therefore, we conclude that spICP-SFMS is not able to provide information on the thickness of the glycopolymers bound to the GNR surface. The most plausible explanation for this observation is that spICP-SFMS measurements are based on single particle detection via the ^197^Au ion signal and that the coating thickness of 1.5–1.8 nm as estimated from DCS data, will not play a role in the GNRs particle size detection. However, from the size distributions it was evident that both short and long glycopolymers did not affect the colloidal stability of GNRs. This information is an important factor in biomedical applications as the localized surface plasmon resonance signal generated by GNRs is shape- and size-dependent [5,6]. Comparing the particle size distribution as obtained for the bare GNRs with those for GNR-Gal-PHEA35 and GNR-Gal-PHEA60, demonstrates that spICP-SFMS is very useful as an orthogonal method for accurate determination of GNRs size distribution, as well as for providing information on possible particle colloidal instability after binding with synthetic glycopolymers [40]. Therefore, the EAF4-MALS method was further applied to examine the thickness of the glycopolymers bound on the GNRs surface.

### 3.2. Characterization of GNRs and Glycoconjugated GNRs Using EAF4-MALS

#### 3.2.1. Optimization of EAF4-MALS Method

Systematic evaluation of the composition of the carrier solution with varying concentrations of sodium nitrate (10 mM, 8 mM, 4 mM, 2 mM, 1 mM, 0.5 mM) showed that the ionic strength, thus also the pH, have a large impact on the GNR recovery. Bare GNRs do not elute at sodium nitrate concentrations of 4 mM or higher. The highest recovery was obtained at 0.5 mM sodium nitrate, the concentration thus used for all further EAF4 runs. The effect of carrier ionic strength and composition on the resolution, recovery, and reproducibility of AF4 fractionation of citrate-stabilized gold nanoparticles has been shown before [41]. In EAF4, the choice of carrier composition is limited, because additives that could form unwanted reaction products via electrode reaction should be avoided. While the recovery of bare GNRs was still low under the optimized conditions, the glycoconjugated GNRs showed much higher recoveries of approximately 85% (Table 5), indicating a changed surface chemistry.

#### 3.2.2. Performance of EAF4-MALS Method

**Figure 4:**
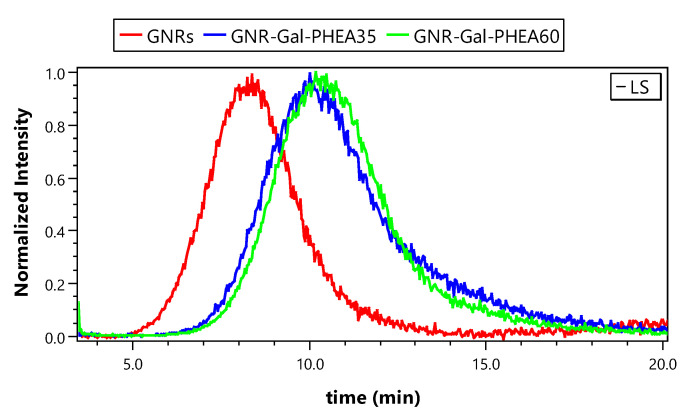
90° light scattering fractograms of GNRs, GNR-Gal-PHEA35 and GNR-Gal-PHEA60.

The size of GNRs was assessed using EAF4-MALS runs without an amperage applied during separation (Figure 4). The retention time could then be directly related to the hydrodynamic size of the particles. We observed an increased retention time of glycoconjugated GNRs compared to the bare GNRs (Figure 4) indicating an increase in hydrodynamic size, which was highest for the longest glycopolymers. In addition, spICP-SFMS results have shown that Au signals of the bare GNRs and glycoconjugated GNRs were not affected. Therefore, the increase in hydrodynamic diameter can be attributed to the glycoconjugation of the GNRs. This confirmed successful coating of GNRs [20]. In addition, orthogonal information obtained from spICP-SFMS (Table 4) and EAF4-MALS (Table 6) can also be used to estimate the coating thickness of glycoconjugated GNRs and the results thus obtained correspond well to the coating thicknesses reported for GNR-Gal-PHEA35 (0.3–0.8 nm) and GNR-Gal-PHEA60 (1.1–1.6 nm) based on the use of DCS (Table 1). As such, the combination of EAF4-MALS with spICP-SFMS presents a reliable approach for characterization of gold nanorods conjugated with synthetic glycopolymers.

The surface charge of GNRs is assessed by comparing EAF4-MALS runs with (−0.1 mA) and without (0.0 mA) an amperage applied during separation. Shifts towards a higher retention time of up to 0.5 min are observed for each sample when applying −0.1 mA. This indicates that GNRs carry a negative charge, both before and after glycoconjugation, confirming the ζ-potential results measured using a ZetaView-Twin instrument (Particle Metrix, Inning am Ammersee, Germany) [20]. However, accurate determination of electrophoretic mobility and zetapotential would require extensive EAF4 runs at 3–4 different amperages.

### 3.3. spICP-SFMS and EAF4-MALS as a Complementary Techniques to DLS, DCS and NTA

As mentioned in the introduction, the characterization of gold nanorods conjugated with synthetic glycopolymers is of high importance. Thus, in this work we used well-characterized GNRs and GNRs conjugated with synthetic glycopolymers in terms of particle size and coating thickness.

The combination of spICP-SFMS and EAF4-MALS as complementary techniques to DLS, NTA and DCS was demonstrated here to be of high value in this context.

All the techniques used here provide the strong evidence that bare-GNRs and gly-coconjugated GNRs are properly dispersed in solution and have been successfully functionalized: the UV-Vis red shift of the LSPR band (Figure S3) is a result of a change of the local refractive index due to glycopolymers binding; DLS, DCS and NTA showed an increase in particle size after polymer addition (Table S1).

**Figure 5:**
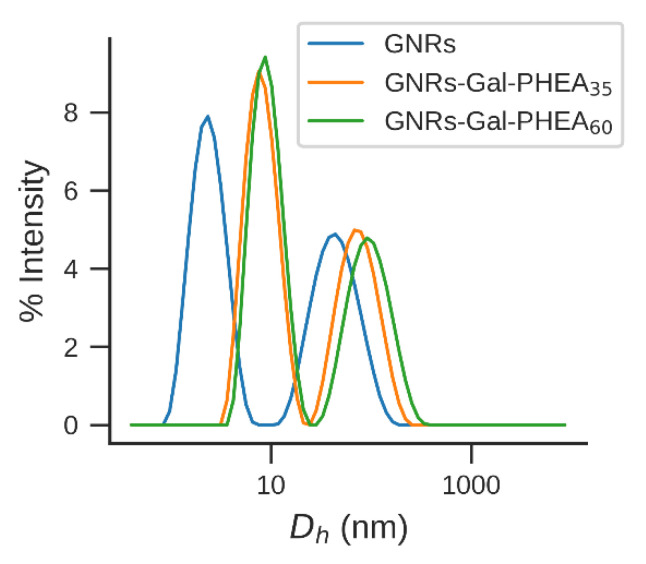
Intensity-weighted DLS size distributions of GNRs-Gal-PHEA35 and GNRs-Gal-PHEA60 compared to bare GNRs.

Dynamic light scattering (DLS) is a widely employed technique for nanoparticle size analysis. DLS measures the diffusion coefficient of the particle dispersed in a colloidal solution, which is dependent on the mass, the shape and the surface chemistry of the particles [42]. These parameters affect the particle–solvent interactions, and therefore, the Brownian motion. DLS generally assumes spherical shaped particles but also non-spherical shapes such as nanorods have been characterized by multiple angles or depolarized DLS measurements [43,44] and fixed angle DLS [45]. The DLS results of the samples showed two peaks (Figure 5): the small-sized peak, usually misinterpreted as the presence of smaller particle impurities, is attributed to the rotational diffusion arising from the GNRs anisotropic shapes [46,47]. It is not an actual dimension of the nanorods and it has been demonstrated to be strongly dependent on the aspect ratio [45]. GNRs, GNRs-Gal-PHEA35 and GNRs-Gal-PHEA60 have a rotational diffusion coefficient equivalent to the translation diffusion coefficient of a spherical particle with an average diameter of 7 nm, 13 nm and 15 nm, respectively, and the same diffusion coefficient as a spherical gold nanoparticle with a hydrodynamic diameter of 49 nm, 81 nm and 105 nm, respectively. Correct interpretation of DLS results for the determination of the size and coating thickness of gold nanorods requires careful analysis of the results obtained.

NTA also measures nanoparticle size distribution of samples in liquid dispersion (Figure S4), at a lower concentration detection limit than DLS. Moreover, while DLS studies an ensemble of particles, NTA tracks single particles. It has been reported that the NTA-determined hydrodynamic size of low-aspect-ratio (3.6) GNRs stabilized with citrate correlate closely with GNR length, with greater accuracy and precision than attainable with DLS [48]. In our case, the increasing size after polymer addition correlated well with UV-Vis LSPR shift.

DCS measures particle size based on its sedimentation rate, which depends on their size and density. While DLS is a lower resolution analysis method, DCS offers a high resolution and can be used to characterize particles within a wide range of sizes (2 nm to 50 μm) and made of any material, the density of which is different from that of the solvent [49,50]. With this method, it is possible to measure non-spherical particles and it can also be used to determine the ligand shell thickness as previously mentioned [38,51]. However, an independent determination of the shape and aspect ratio of nanorods is required. Although DCS is reported to be more precise than either DLS or NTA, and is less prone to artifacts, in some cases it may underestimate the coating shell thickness [52].

The combined use of different techniques could yield significant insights regarding size and coating thickness of the gold nanorods studied.

## 4. Conclusions

A comprehensive characterization of GNRs conjugated with synthetic glycopolymers presents an added value in biomedical applications. Therefore, development of complementary analytical techniques that can provide reliable information on particle size, shape, number concentration and coating thickness is crucial. The current study achieved its main goals concerning the characterization of the GNRs conjugated with short and long synthetic glycopolymers by using a combination of spICP-SFMS and EAF4-MALS. The GNRs were separated and characterized via EAF4-MALS on the basis of size and surface charge, while spICP-SFMS provided information on the particle number concentration, size and size distribution. In addition, EAF4-MALS appeared to be suitable for estimating the coating thickness of glycoconjugated GNRs. Finally, knowing that a universal analytical method for particle characterization still does not exist, further research is needed to prove the significant advantage offered by joining the capabilities of spICP-SFMS and EAF4-MALS (e.g., reproducibility) and possibly AF4-UV/VIS-fluorescence-MALS-ICP-MS for the characterization of GNRs conjugated with synthetic glycopolymers when compared to more common characterization methods, such as UV-VIS and DCS.

## Figures and Tables

**Table 1:** Characteristics of the GNRs, GNR-Gal-PHEA35 and GNR-Gal-PHEA60.

	Peak Size (nm)	Calculated Coating Thickness (nm)
**GNRs ^a^**	22.0 ± 0.1	n/a
**GNR-Gal-PHEA35 ^b^**	19.7 ± 0.1	1.5 ± 0.1
**GNR-Gal-PHEA60**	19.2 ± 0.1	1.8 ± 0.1

^a,b^ Particle size distribution measured using DCS as previously reported by Pancaro et al. 2021, [20]. N = 2, mean ± SD.

**Table 2:** ICP-MS instrument settings and data acquisition parameters.

Parameter	
Radio frequency power	1300 W
Plasma gas flow rate	13 L min^−1^
Carrier gas flow rate	0.93 L min^−1^
Measurement mode	TRA
Nuclide monitored	^197^Au
Dwell time	40 µs
Acquisition time	60 s
Nebulizer	MicroMist
Spray chamber	Cyclonic

Data acquisition and data treatment were performed using the combination of NuAttoLab and NuQuant sofware (Nu Instruments, Wrexham, UK).

**Table 3:** Settings for EAF4 Separation.

Parameter	
Tip to tip channel length	26.5 cm
Spacer	350 μm
Focus flow rate	1 mL min^−1^
Injection flow rate	0.2 mL min^−1^
Injection time	3 min
Focus time	2 min
Elution time	30 min
Detector flow rate	1 mL min^−1^
Cross-flow rate	0.5 mL min^−1^
Membrane	PES, 5 kDa
Carrier	0.5 mM sodium nitrate

**Table 4:** spICP-SFMS results of bare and glycoconjugated GNRs (N = 2, mean +/− SD).

	Spherical Equivalent Diameter (nm)	Particle Number Concentration (Particles per mL)
**GNRs**	21.0 ± 0.5	1.83 × 10^7^ ± 1.30 × 10^6^
**GNR-Gal-PHEA35**	21.0 ± 0.4	4.78 × 10^7^ ± 1.10 × 10^6^
**GNR-Gal-PHEA60**	22.0 ± 0.0	5.27 × 10^7^ ± 4.29 × 10^6^

**Table 5:** Recovery of bare and glycoconjugated GNRs. The data are calculated from AF4-MALS fractograms.

	GNRs	GNR-Gal-PHEA35	GNR-Gal-PHEA60
**Injected Mass (µg)**	0.84	0.06	0.06
**LS peak area Flow Through** **(cm^−1^ min^−1^)**	2.617 × 10^−5^	1.053 × 10^−5^	1.443 × 10^−5^
**LS peak area Separation run** **(cm^−1^ min^−1^)**	4.431 × 10^−6^	8.786 × 10^−6^	1.242 × 10^−6^
**Recovery (%)**	16.9	83.4	86.1

**Table 6:** Hydrodynamic diameter D_h_ determined via EAF4-MALS.

	GNRs	GNR-Gal-PHEA35	GNR-Gal-PHEA60
D_h_ (nm)	17.0	21.3	22.1

## Data Availability

The data presented in this study are available on request from the corresponding author.

## Supplementary Materials

The following Supplementary Materials are available online at https://www.mdpi.com/article/10.3390/nano11102720/s1, Synthetic Method S1. Photo-polymerization of N-(2-hydroxyethyl) acrylamide (HEA) via photo-initiated RAFT and end-group modification of PFP-poly(N-hydroxyethyl acrylamide) (PFP-PHEA) homopolymers using galactosamine. Figure S1. Differential centrifugal sedimentation analysis results for GNRs (blue line), GNRs-Gal-PHEA35 (orange line) and GNRs-Gal-PHEA60 (green line). Representative examples out of three replicates of relative weight as a function of particle diameter (micrometer). Inset: zoomed view of the peaks, Figure S2. Representative dry-state TEM image of GNRs-Gal-PHEA35, Figure S3. Representative example of UV-Vis absorption spectra for GNRs (blue line), GNRs-Gal-PHEA35 (orange line) and GNRs-Gal-PHEA60 (green line). Inset: zoomed view on the LSPR peak bands, Figure S4. Particle number-based size distribution for GNRs (blue line), GNRs-Gal-PHEA35 (orange line) and GNRs-Gal-PHEA60 (green line) as determined by NTA, Table S1. Characterization of Glycopolymer-coated GNRs. UV-Vis LSPR peak (nm), ζ-potential (mV), and peak diameter (nm) by DCS and mode (nm) by NTA for citrate-GNRs and glycopolymer-coated GNRs.
