# Supplementary material for: Characterization of Gold Nanorods Conjugated with Synthetic Glycopolymers Using an Analytical Approach Based on spICP-SFMS and EAF4-MALS
Source: Nanomaterials (Basel). 2021 Oct 15;11(10):2720. doi: 10.3390/nano11102720 (PMC8539460; doi:10.3390/nano11102720)
Supplement: Supplementary file 1 [file nanomaterials-11-02720-s001.zip › nanomaterials-1408812-supplementary.pdf]

nanomaterials

Supplementary Materials

Characterization of Gold Nanorods Conjugated with Synthetic Glycopolymers Using an Analytical Approach Based on spICP-SFMS and EAF4-MALS

Milica Velimirovic [1,2,*], Alessia Pancaro [2,3], Robert Mildner [4], Panagiotis G. Georgiou [5], Kristof Tirez [2], Inge Nelissen [2], Christoph Johann [4], Matthew I. Gibson [5,6] and Frank Vanhaecke [1]

1 Department of Chemistry, Atomic & Mass Spectrometry–A&MS Research Group, Campus Sterre, Ghent University, Krijgslaan 281-S12, 9000 Ghent, Belgium; frank.vanhaecke@ugent.be
2 Flemish Institute for Technological Research (VITO), Boeretang 200, 2400 Mol, Belgium; alessia.pancaro@vito.be (A.P.); kristof.tirez@vito.be (K.T.); inge.nelissen@vito.be (I.N.)
3 Advanced Optical Microscopy Centre and Biomedical Research Institute, Hasselt University, 3590 Diepenbeek, Belgium
4 Wyatt Technology Europe GmbH, Hochstrasse 12a, 56307 Dernbach, Germany; Robert.Mildner@wyatt.eu (R.M.); cjohann@wyatt.eu (C.J.)
5 Department of Chemistry, University of Warwick, Gibbet Hill Road, Coventry CV4 7AL, UK; P.Georgiou@warwick.ac.uk (P.G.G.); M.I.Gibson@warwick.ac.uk (M.I.G.)
6 Warwick Medical School, University of Warwick, Gibbet Hill Road, Coventry CV4 7AL, UK
* Correspondence: milica.velimirovic@vito.be; Tel.: +32-14-335-779

Synthetic Method S1. Photo-polymerisation of N-(2-hydroxyethyl) acrylamide (HEA) via photo-initiated RAFT and end-group modification of PFP-poly(N-hydroxyethyl acrylamide) (PFP-PHEA) homopolymers using galactosamine.

The following procedure describes a reaction for [monomer]:[PFP-DMP] ratio of 140 and 180. N-(2-hydroxyethyl) acrylamide (HEA) (1.52 g, 13.2 mmol and 1.95 g, 17 mmol, respectively) and raft agent of 2-(dodecylthiocarbonothiolythio)-2-methylpropanoic acid pentafluorophenyl ester (PFP-DMP) (0.05 g, 0.094 mmol) were dissolved in 50:50 dioxane:methanol solution (6.27 mL and 8.01 mL, respectively) in a vial. The resulting solutions were degassed by sparging N_2(g) for 15 min and the sealed vials were incubated at 37 °C with magnetic stirring under 460 nm light irradiation for 120 min. The reactions were rapidly cooled and precipitated into diethyl ether from methanol three times to yield two yellow polymers products which were further dried under vacuum. An aliquot of crude polymerisation mixture was taken for ^{1}H NMR in methanol-d_4 for conversion and M_n, NMR analysis. ^{1}H, ^{19}F NMR and SEC analysis were conducted on purified polymers after precipitation. Conversions were calculated using ^{1}H NMR spectroscopy by comparing the integrations of the HEA monomer signal (δ 5.67 ppm) with this of the C*H* of the PHEA backbone (δ 2.22-2.04 ppm). $M_{n,\ NMR}$ was calculated by end-group analysis, i.e., by comparing the integration of the -CH3 signal (δ 0.92 ppm) of the dodecyl end-group with this of the C*H* of the PHEA backbone (δ 2.22-2.04 ppm). ^{1}H NMR (400 MHz. methanol-d_4): δ (ppm) 8.15-8.03 (br m, N*H* of PHEA side chain), 3.89-3.13 (br m, NH-CH_2 and CH_2-OH of PHEA side chain), 2.35–2.05 (br m, C*H* of PHEA backbone), 1.85-1.31 (br m, CH_2 of PHEA backbone), 0.92 (t, 3H, CH_2-CH_3 of dodecyl end-group). FT-IR (neat): ν (cm^{-1}) 3300 (N-H and O-H stretch); 2868 (alkyl C-H stretch); 1772 (C_6F_5C=O stretch); 1638 (amide C=O stretch); 1544 (N-H bend); 1438 (C-H bend); 1216 (C-O stretch); 1060 (C-O stretch); 950 (C-F peak on shoulder of 1060 peak).

PFP-PHEA35 and PFP-PHEA60 (50 mg, 0.011 mmol and 50 mg, 0.007 mmol, respectively), were dissolved in 5 mL DMF with 0.05 M triethylamine (TEA) (50 μL) with galactosamine (11.7 mg, 0.054 mmol and 7.25 mg, 0.034 mmol, respectively). The reactions were stirred at 50 °C for 16 hrs. The polymers were precipitated into diethyl ether from

methanol three times and dried over under vacuum. ^{19}F-NMR and IR analysis were performed and confirmed the loss of the pentafluoro end-group for both polymers.

Polymers characterization. Polymers synthesised by photo-RAFT.

Polymer[a] (-)	[M]:[CTA][b] (-)	Conversion (%)[c]	$M_{n, NMR}$ (g mol^{-1})[d]	*SEC analysis*	
				$M_{n, SEC}$ (g mol^{-1})[e]	$Đ_M$ [e]
$PHEA_{35}$	140	25	4600	9800	1.12
$PHEA_{60}$	180	33	7400	16500	1.11

[a] Polymer names are determined according to the average degree of polymerisation (DP) determined by ^{1}H-NMR end-group analysis in methanol-d_4. [b] [M]:[CTA] indicates the ratio between monomers (M) and the chain transfer agents (CTA). [c] Monomer conversion (%) calculated by comparing the integration values of the monomer signals with those of the corresponding signals of the polymer. [d] $M_{n, NMR}$ was calculated by end-group analysis by comparing the integrations of the –CH_3 signals (δ 0.92 ppm) of dodecyl end-group with those of the corresponding signals of the polymer backbone. [e] M_n and $Đ_M$ values calculated from PMMA standards using 5 mM NH_4BF_4 in DMF as the eluent.

Table S1. Characterization of Glycopolymer-coated GNRs. UV-Vis LSPR peak (nm), ζ-potential (mV), and peak diameter (nm) by DCS and mode (nm) by NTA for citrate-GNRs and glycopolymer-coated GNRs.

	UV-Vis LSPR peak (nm)	ζ-Potential (mV)	DCS Peak diameter (nm)	NTA size Mode (nm)
**GNRs**	779 ± 1	- 44.9 ± 2.3	22.3 ± 0.1	42.8 ± 1.3
**GNRs-Gal-PHEA$_{35}$**	787 ± 1	- 33.8 ± 1.8	19.7 ± 0.1	52.4 ± 1
**GNRs-Gal-PHEA$_{60}$**	788 ± 1	- 33.7 ± 1.3	19.2 ± 0.1	53.7 ± 0.6

After glycopolymer functionalisation, the UV-Vis LSPR peak showed a red shift and the absolute value of the negative ζ-potential decreased. NTA, DLS and DCS indicated an increased in particle size, confirming the successful attachment of the glycopolymers to the particle surface. The mean ± SD of three replicates of UV-Vis, ζ-potential, DCS and NTA measurements are shown.

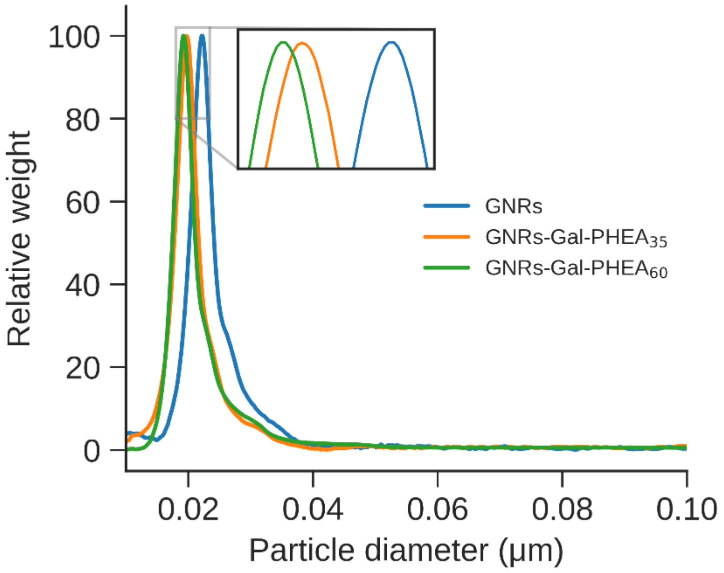

Figure S1. Differential centrifugal sedimentation analysis results for GNRs (blue line), GNRs-Gal-$PHEA_{35}$ (orange line) and GNRs-Gal-$PHEA_{60}$ (green line). Representative examples out of three replicates of relative weight as a function of particle diameter (micrometer). Inset: zoomed view of the peaks.

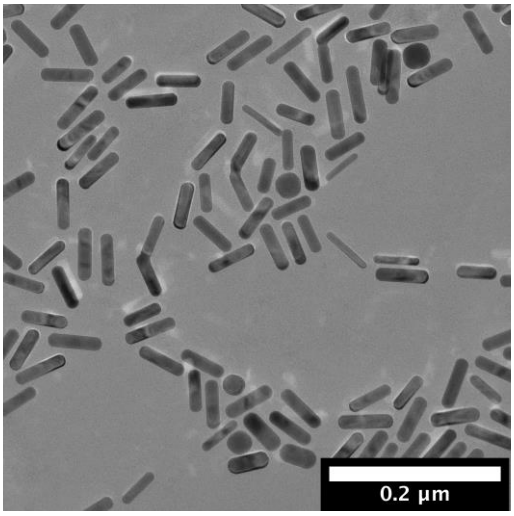

Figure S2. Representative dry-state TEM image of GNRs-Gal-PHEA35.

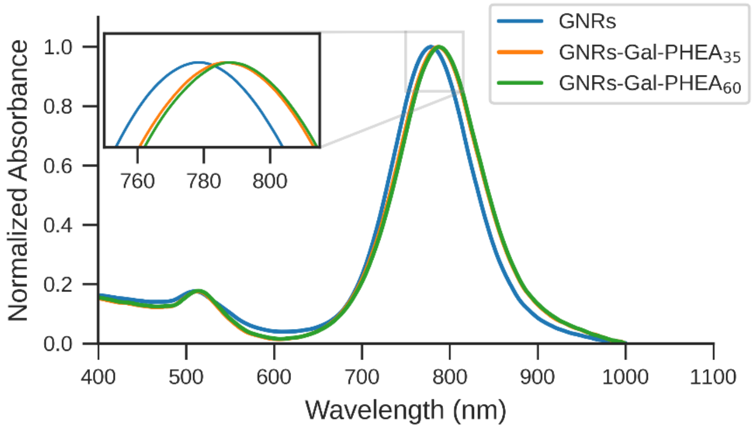

Figure S3. Representative example of UV-Vis absorption spectra for GNRs (blue line), GNRs-Gal-PHEA35 (orange line) and GNRs-Gal-PHEA60 (green line). Inset: zoomed view on the LSPR peak bands.

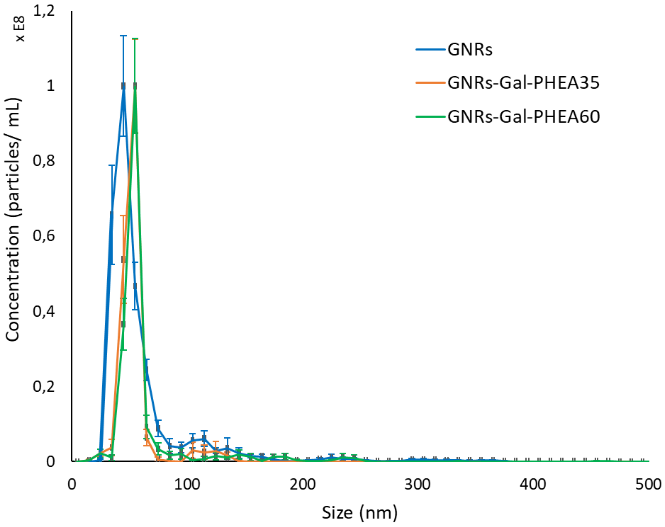

Figure S4. Particle number-based size distribution for GNRs (blue line), GNRs-Gal-PHEA35 (orange line) and GNRs-Gal-PHEA60 (green line) as determined by NTA.
